# The Effect of Live-Virus Vaccines on Tests for Tuberculosis Infection During the US Immigration Medical Examination: Are Vaccines Causing False-Negative Results?

**DOI:** 10.1093/cid/ciaf129

**Published:** 2025-05-15

**Authors:** Joanna J Regan, Zanju Wang, Christina R Phares

**Affiliations:** Division of Global Migration Health, Centers for Disease Control and Prevention, Atlanta, Georgia, USA; Division of Global Migration Health, Centers for Disease Control and Prevention, Atlanta, Georgia, USA; Division of Global Migration Health, Centers for Disease Control and Prevention, Atlanta, Georgia, USA

**Keywords:** tuberculosis, immigration, screening, live-virus vaccines, latent tuberculosis infection (LTBI)

## Abstract

**Background:**

It is not recommended to perform tuberculin skin tests (TSTs) or interferon-γ release assays (IGRAs) in the 4 weeks following live-virus vaccination because these vaccines are thought to increase the risk of false-negative results.

**Methods:**

We retrospectively analyzed TST and IGRA results for 158 484 US-bound immigrant and refugee children aged 2–14 years who received a required medical examination and live-virus vaccines (measles, mumps, rubella; oral polio; or varicella) overseas during 2014–2022. We created logistic regression models to assess the association between test positivity and vaccination during the critical interval (1–28 days after live-virus vaccination) versus after or before, adjusting for sex, age group, country of examination, and other factors.

**Results:**

The percentage of positive results and the adjusted odds of a positive IGRA result were higher for children tested during the critical interval (4.6%) than for those tested after (3.5%) (adjusted odds ratio, 1.27 [95% confidence interval, 1.13–1.43) or before (3.3%) (1.26 [1.13–1.41]). The percentage of positive results and the adjusted odds of a positive TST were also higher for children tested during the critical interval (15.7%) than for those tested after (7.2%) (adjusted odds ratio, 2.40 [95% confidence interval, 1.79–3.22]) or before (6.6%) (3.81 [2.80­–5.18]).

**Conclusions:**

The concern that recent administration of live-virus vaccines leads to false-negative TST and IGRA results is not supported by these findings. Instead, we observed a modest increase in positive results among children tested during the critical postvaccination interval, challenging the need for the testing delay.

## Teaser

Live-virus vaccines are thought to suppress the response to tuberculosis infection tests for 4 weeks, increasing the risk of false-negative results. This analysis found instead a small increase in positive results postvaccination and challenges the need for testing delays.

In the early 1900s, infection with the wild-type measles virus was found to induce immunosuppression, and studies since have demonstrated that this effect can be life threatening [1, 2]. Immunosuppression caused by measles and other viral infections was studied through tuberculin skin test (TST) reaction depression, although it is unclear whether clinically significant immunosuppression and the phenomenon of TST reaction depression were caused by the same mechanism [1, 3–5]. Later, live-virus vaccines were associated with TST reaction depression [4–7], and some early preparations of attenuated measles virus–containing vaccines appeared to cause clinically significant immunosuppression similar to wild-type virus infection [8, 9].

These findings led to the recommendation to delay TST for ≥4 weeks if a live-virus vaccine had been administered, particularly the measles-mumps-rubella (MMR) vaccine [10–12]. In 1975 a clarification was made to allow for TST testing to be performed on the same day as the administration of live-virus vaccines [13]. Although studies demonstrated that clinically significant immunosuppression is not experienced by recipients of modern measles virus–containing vaccines [2, 14], this recommendation has persisted for decades, often citing theoretical concerns. This recommendation has been expanded to include interferon-γ release assays (IGRA) for detection of tuberculosis infection [10, 12], although to our knowledge no studies of the effects of live-virus vaccines on IGRA results have been published.

All immigrants and refugees applying for admission to the United States must be evaluated for tuberculosis disease according to the Tuberculosis Technical Instructions for Panel Physicians [15]. All children ages 2–14 years old screened in countries in which the estimated tuberculosis incidence rate is ≥20 cases per 100 000 people per year must undergo IGRA testing (TST could be used prior to 2018) before arriving in the United States. Because the rates of active tuberculosis disease in children are lower than that in adults [16], applicants ≥15 years of age are required to undergo chest radiography rather than a TST or IGRA [15]. Immigrants are required to receive certain vaccines before immigrating, and the delay before testing with IGRA or TST after live-virus vaccination was never required for immigrant children. However, panel physicians have requested evidence to ensure that testing during this interval is not leading to false-negative results. Refugees are required to receive applicable vaccinations in outbreak settings but otherwise are offered voluntary vaccinations before resettling, through the Vaccination Program for US-Bound Refugees, and the 4-week delay was recommended for this program by the Division of Global Migration Health of the US Centers for Disease Control and Prevention (CDC). This delay can cause a cascade of processing issues that may postpone travel by months or years. This recommendation can also cause missed immunization opportunities, as vaccines are typically not offered in the last 28 days before departure to avoid delays in tuberculosis evaluation on arrival in the United States [17].

This 4-week wait is a problem for immigrants and refugees. Thus, we sought to determine how observing versus not observing this delay might be affecting TST and IGRA results. We analyzed existing observational data to determine whether children who received an IGRA or TST within 1–28 days after live-virus vaccination as part of the overseas medical examination were less likely to test positive than those who received their IGRA or TST on the same day as vaccination or >28 days after vaccination. This evaluation will inform the US immigration tuberculosis screening requirements.

## METHODS

### Analysis Population

We assessed the data for immigrant and refugee children aged 2–14 years who received live-virus vaccines overseas and had an IGRA or TST performed as part of their overseas medical examination in 2014‒2022. Data for refugee children were available for all years; data for immigrant children were available for 2018‒2022. Although the brand of IGRA or TST was not captured in this dataset, the Tuberculosis Technical Instructions for 2014–2018 stated that “Purified protein derivative (PPD) should be administered intradermally by the Mantoux method. Ideally, preparations used should be equivalent to 5 tuberculin units PPD-S. However, in countries where such preparations are limited or impossible to import, panel physicians should use PPD preparations that are approved for use by their ministries of health.” On 1 October 2018, these instructions were updated to require IGRA instead of TST testing, specifying that US Food and Drug Administration–approved IGRAs must be used; these included QIAGEN QuantiFERON (any iteration approved by the Food and Drug Administration) or Oxford Immunotec T-SPOT. Although these IGRA tests were allowed before 2018, the primary testing method was TST until IGRA testing became a requirement [15].

### Definitions

Live-virus vaccines were divided into 4 categories (1) measles, mumps, rubella vaccine, which included any vaccine containing ≥1 of these 3 viral components; (2) oral polio vaccine (OPV); (3) varicella vaccine; and (4) any live-virus vaccine, which included any vaccine from the first 3 categories. Panel physicians are required to record all valid vaccine information provided, including historical doses given by other providers [18]. Children whose vaccination records lacked an administration date were excluded.
For IGRA results, we used the IGRA interpretation as recorded by the examining physician, which was interpreted according to the package insert. Children whose records had missing or inconclusive results or lacked a test date were excluded. For TST results, we used millimeters of induration as recorded by the physician. Induration ≥10 mm was considered positive except in children with known exposure to tuberculosis disease, (ie, known tuberculosis contacts), for whom ≥5 mm induration was considered positive. Children whose records lacked a test date were excluded.
For the timing of IGRA/TST relative to live-virus vaccination, all live-virus vaccines and IGRA/TST in this analysis were administered overseas. Vaccination in each of the 4 vaccine categories for each child was used to calculate timing relative to the *critical interval* for IGRA/TST, defined as 1–28 days after the last live-virus vaccination administered before the day of the test. If the last live-virus vaccination was 1–28 days before the test, the child was tested *during* the critical interval. If the last live-virus vaccination was >28 days before the test, the child was tested *after* the critical interval. If the last live-virus vaccination occurred on the day of the test, the child was tested *before* the critical interval.
For immigrant children, we used the country of birth as the country of origin. For refugee children, we used the country of nationality. The country of examination was the country where overseas immigration medical examination was conducted.

### Data Sources

We used data from the CDC’s Electronic Disease Notification [19] system for refugee children who were examined overseas from 2014–2022 supplemented with demographic data (nationality) from US Department of State's Worldwide Refugee Admissions Processing System (WRAPS). We used data from CDC's eMedical system, launched in 2018, for immigrant children who were examined overseas during 2018–2022. For all sources, IGRA data for children from Ukraine were excluded because of poor quality of the IGRA results produced by the laboratory used during the analysis period.

### Statistical Analysis

We summarized arrival dates and demographic characteristics of immigrant and refugee children who had received any live-virus vaccines. We conducted χ^2^ tests to compare the proportion with a positive IGRA/TST result who were tested before, during, or after the critical interval for each vaccine category.

To assess the association between test positivity and test timing relative to vaccination with any live-virus vaccine, we created separate IGRA and TST logistic regression models. Both models adjusted for sex, age group, country of examination, and status as known tuberculosis contact. Countries of examination where <20 children had a positive test result (for the test being modeled) were grouped together. The IGRA model also adjusted for immigrant versus refugee status. Because data for immigrant children were not available for 2014–2017 when the primary testing method was TST, immigrant children were excluded from the TST model. In both models, test timing was included as a single categorical variable with 3 mutually exclusive levels corresponding to children tested before, during, or after the critical interval. SAS software (version 9.4; SAS Institute) was used for statistical analysis.

### Ethics Review

This activity was reviewed by the CDC, deemed not research, and conducted consistent with applicable federal law and CDC policy (see, eg, 45 CFR part 46; 21 CFR part 56; 42 USC §241[d]; 5 USC §552a; and 44 USC §3501 et seq).

## RESULTS

After excluding 1774 children (0.89%) due to missing or indeterminate test results or missing dates, the analysis included the remaining 158 484 children who received ≥1 live-virus vaccine overseas and were tested by means of IGRA (125 329) or TST (33 155) before, during, or after the critical period. Among all 158 484 children, 22% were aged 2–4 years, 39% aged 5–9 years, and 39% aged 10–14 years; 51% were male. For 125 329 children tested with IGRA, 86% were immigrants and 14% were refugees, and almost all (97%) were examined during 2019–2022 (Table 1). For 33 155 children tested with TST, all were refugees, and almost all (99%) were examined during 2014–2018 (Table 2). The rate of active tuberculosis disease in these children is 30 cases per 100 000 children screened.

**Table 1.** Characteristics of US-Bound Immigrant and Refugee Children Who Received Any Live-Virus Vaccine and Were Tested With an Interferon-γ Release Assay During the Overseas Medical Examination^a^

Characteristics	Children, No. (%)
Year of examination	
2014	264 (0.2)
2015	1277 (1.0)
2016	755 (0.6)
2017	163 (0.1)
2018	1856 (1.5)
2019	12 415 (9.9)
2020	14 665 (11.7)
2021	44 933 (35.9)
2022	49 001 (39.1)
Sex	
Male	64 177 (51.2)
Female	61 152 (48.8)
Age at examination	
2-4 y	23 725 (18.9)
5-9 y	48 791 (38.9)
10-14 y	52 813 (42.1)
Country of origin^b^	
Dominican Republic	15 905 (12.7)
Mexico	12 722 (10.2)
Afghanistan	8953 (7.1)
China	8091 (6.5)
Vietnam	7566 (6.0)
Other	71 380 (57.0)
Unknown	712 (0.6)
Country of examination^b^	
Dominican Republic	16 038 (12.8)
Mexico	12 749 (10.2)
China	8408 (6.7)
Vietnam	7582 (6.0)
Guyana	5245 (4.2)
Other	75 307 (60.1)
Visa type	
Immigrant	107 998 (86.2)
Refugee	17 331 (13.8)
Total	125 329 (100.0)

^a^Children were aged 2–14 years and were vaccinated with ≥1 of the following: vaccine containing ≥1 of measles, mumps, or rubella vaccine; oral polio vaccine; or varicella vaccine.

^b^The top 5 countries of origin and examination are listed, with the remaining countries grouped as “other.”

**Table 2.** Characteristics of US-Bound Refugee Children Who Received Any Live-Virus Vaccination and Were Tested With a Tuberculin Skin Test During the Overseas Medical Examination^a^

Characteristic	Children, No. (%)
Year of examination	
2014	6673 (20.1)
2015	7944 (24.0)
2016	11 026 (33.3)
2017	3971 (12.0)
2018	3282 (9.9)
2019	136 (0.4)
2020	46 (0.1)
2021	54 (0.2)
2022	23 (0.1)
Sex	
Male	16 937 (51.1)
Female	16 218 (48.9)
Age at examination	
2-4 y	10 834 (32.7)
5-9 y	12 672 (38.2)
10-14 y	9649 (29.1)
Country of origin^b^	
Burma	11 369 (34.3)
Democratic Republic of the Congo	7485 (22.6)
Somalia	4888 (14.7)
Bhutan	4586 (13.8)
Eritrea	791 (2.4)
Other	4029 (12.2)
Unknown	7 (0.0)
Country of examination^b^	
Malaysia	7583 (22.9)
Nepal	4632 (14.0)
Thailand	4096 (12.4)
Tanzania	3598 (10.9)
Kenya	3387 (10.2)
Other	9859 (29.7)
Visa type	
Refugees	33 155 (100.0)
All	33 155 (100.0)

^a^Children were aged 2–14 years and were vaccinated with ≥1 of the following: vaccine containing ≥1 of measles, mumps, or rubella vaccine; oral polio vaccine; or varicella vaccine.

^b^The top 5 countries of origin and examination are listed, with the remaining countries grouped as “other.”

### Proportion of Children Tested Before, During, and After the Critical Interval

For children tested with IGRA, 9% were tested during the critical interval (1–28 days following any live-virus vaccine) versus 55% before the interval (same day as any live-virus vaccine) and 36% after it (≥29 days after any live-virus vaccine). For children tested during the critical interval, a mean and median of 4 and 7 days, respectively, elapsed between vaccination and IGRA (25th and 75th percentiles, 2 and 9 days). For those tested with TST, 1% were tested during the critical interval versus 39% before and 60% after. For those tested during the critical interval, the time elapsed between vaccination and TST was a mean of 11 and a median 7 days (25th and 75th percentiles, 3 and 21 days).

### Use of χ^2^ Tests to Compare Proportions of Children Testing Positive

The proportion of children with a positive IGRA result was similar for children tested before or after the critical interval for each type of vaccine (range, 3.0%–3.6%); for children tested during the critical interval, the proportion who were IGRA positive varied by vaccine type, ranging from 4.9% following varicella vaccine to 4.1% following measles, mumps, rubella vaccine to 2.7% following OPV, but the proportion of children with a positive IGRA result was higher for children tested during the critical interval than for those tested before or after it for each type of vaccine except OPV (*P* < .001) (Table 3). The proportion of children with a positive TST result varied across vaccine types. However, for each vaccine type, the proportion who were TST positive was higher among children tested during the critical interval (range, 14.2%–18.2%) than among those tested before (range, 1.6%–7.1%) or after (5.4%–9.6%) (*P* < .001) (Table 4).

**Table 3.** Interferon-γ Release Assay Positivity for US-Bound Immigrant and Refugee Children Tested Before, During, or After the Critical Interval for Live-Virus Vaccination

Live-Virus Vaccines Administered Overseas	Timing of IGRA Overseas Relative to Last Live-Virus Vaccine Received Overseas^a^						
	Before Critical Interval (Same Day)		During Critical Interval (Within 1–28 d)		After Critical Interval (After ≥29 d)		*P* Value
	Total No.	Positive TST Result, No. (%)	Total No.	Positive TST Result, No. (%)	Total No.	Positive TST Result, No. (%)	
Measles, mumps, rubella^b^	47 481	1553 (3.3)	8295	341 (4.1)	63 496	2270 (3.6)	<.001
OPV	4583	149 (3.3)	3001	82 (2.7)	59 161	1957 (3.3)	.22
Varicella	50 003	1751 (3.5)	7059	344 (4.9)	26 816	814 (3.0)	<.001
Any live-virus vaccine^c^	68 678	2246 (3.3)	11 046	512 (4.6)	45 605	1573 (3.5)	<.001

Abbreviation: IGRA, interferon-γ release assay; OPV, oral polio vaccine.

^a^For a given vaccine, the critical interval is defined as 1–28 days following the last vaccination administered before the IGRA. If the IGRA occurred within this window, the child was tested during the critical interval. If the IGRA occurred >28 days after the last vaccination, the child was tested after the critical interval. If the last vaccination occurred on the same day as the IGRA, the child was tested before the critical interval. Testing during the critical interval is not historically recommended.

^b^Vaccine containing ≥1 of measles, mumps, or rubella vaccine.

^c^Vaccination with ≥1 of the following: vaccine containing ≥1 of measles, mumps, or rubella vaccine; OPV; or varicella vaccine.

**Table 4.** Tuberculin Skin Test Positivity for US-Bound Refugee Children Tested Before, During, or After the Critical Interval for Live-Virus Vaccination

Live-Virus Vaccine Received Overseas	Timing of TST Overseas Relative To Last Live-Virus Vaccines Received Overseas^a^						
	Before Critical Interval (Same Day)		During Critical Interval (Within 1–28 d)		After Critical Interval (After ≥29 d)		*P* Value
	Total No.	Positive TST Result, No. (%)	Total No.	Positive TST Result, No. (%)	Total No.	Positive TST Result, No. (%)	
Measles, mumps, rubella^b^	11 363	805 (7.1)	308	56 (18.2)	18 683	1404 (7.5)	<.001
OPV	3615	141 (3.9)	176	25 (14.2)	15 315	829 (5.4)	<.001
Varicella	619	10 (1.6)	7	1 (14.3)	239	23 (9.6)	<.001
Any live-virus vaccine^c^	12 781	838 (6.6)	414	65 (15.7)	19 960	1436 (7.2)	<.001

Abbreviation: OPV, oral polio vaccine; TST, tuberculin skin test.

^a^For a given vaccine, the critical interval is defined as 1–28 days following the last vaccination administered before the TST. If the TST occurred within this window, the child was tested during critical interval. If the TST occurred >28 days after the last vaccination, the child was tested after the critical interval. If the last vaccination occurred on the same day as the TST, the child was tested before the critical interval. Testing during the critical interval is not historically recommended.

^b^Vaccine containing ≥1 of measles, mumps, or rubella vaccine.

^c^Vaccination with ≥1 of the following: vaccine containing ≥1 of measles, mumps, or rubella vaccine; OPV; or varicella vaccine.

### Adjusted Odds of Testing Positive

With the IGRA model, when children tested after the critical interval comprised the reference group for the test timing variable, the adjusted odds of having a positive IGRA result were significantly higher for children tested during the critical interval (adjusted odds ratio [aOR], 1.27 [95% confidence interval (CI), 1.13–1.43]) (Table 5). In a second run of the same model (not shown), now using children tested before the critical interval as the reference group, the odds of having a positive IGRA result were again significantly higher for children tested during the critical interval (aOR, 1.26 [95% CI, 1.13–1.41]). In the TST model, the adjusted odds of having a positive TST were also significantly higher for children tested during the critical interval relative to children tested after it (aOR, 2.40 [95% CI, 1.79–3.22]) (Table 6), and, in a second run (not shown), relative to those tested before the interval (3.81 [2.80–5.18]). The adjusted odds of a positive test were significantly lower among children tested before the critical interval than among those tested after it with the TST model (0.63 [.55–.72) but not the IGRA model (1.00 [.93–1.10).

**Table 5.** Factors Associated With Interferon-γ Release Assay Positivity for US-Bound Immigrant and Refugee Children Tested Before, During, or After the Critical Interval

Variable	Overseas IGRA (N = 125 329)		Univariate Analysis: Crude OR (95% CI)	Multivariate Analysis^a^: Adjusted OR (95% CI)
	Positive IGRA Result, No. (%)	Negative IGRA Result, No. (%)		
Sex				
Male	2182 (3.4)	61 995 (96.6)	Reference	Reference
Female	2149 (3.5)	59 003 (96.5)	1.04 (.97–1.10)	1.03 (.97–1.10)
Age at examination				
2–4 y	494 (2.1)	23 231 (97.9)	Reference	Reference
5–9 y	1407 (2.9)	47 384 (97.1)	1.40 (1.26–1.55)	1.44 (1.30–1.60)
10–14 y	2430 (4.6)	50 383 (95.4)	2.27 (2.06–2.50)	2.44 (2.21–2.70)
Visa type				
Immigrants	3642 (3.4)	104 356 (96.6)	Reference	Reference
Refugees	689 (4.0)	16 642 (96.0)	1.19 (1.09–1.29)	1.01 (.87–1.18)
Tuberculosis contact				
Yes	14 (13.6)	89 (86.4)	4.41 (2.51–7.75)	3.31 (1.86–5.89)
No	4317 (3.5)	120 909 (96.6)	Reference	Reference
IGRA timing relative to vaccination with any live-virus vaccine^b^				
Before critical interval	2246 (3.3)	66 432 (96.7)	0.95 (.89–1.01)	1.00 (.93–1.10)
During critical interval	512 (4.6)	10 534 (95.4)	1.36 (1.23–1.51)	1.27 (1.13–1.43)
After critical interval	1573 (3.5)	44 032 (96.6)	Reference	Reference

Abbreviations: CI, confidence interval; IGRA, interferon-γ release assay; OR, odds ratio.

^a^The multivariate model includes all 5 variables shown in the table plus country of examination, not shown. Countries where <20 people had a positive IGRA result were grouped together, collapsing the country classification to “other” for 4% of individuals.

^b^Vaccination with ≥1 of the following: vaccine containing ≥1 of measles, mumps, or rubella vaccine; oral polio vaccine; or varicella vaccine. For a given vaccine, the critical interval is defined as 1–28 days following the last vaccination administered before the IGRA. If the IGRA occurred within this window, the child was tested during the critical interval. If the IGRA occurred >8 days after the last vaccination, the child was tested after the critical interval. If the last vaccination occurred on the same day as the IGRA, the child was tested before the critical interval. Testing during the critical interval is not historically recommended. This IGRA model used the “after” group as the reference for the IGRA timing variable; when the “before” group is used as the reference, the adjusted OR for children vaccinated during the critical interval is 1.26 (95% CI, 1.13–1.41).

**Table 6.** Factors Associated With Tuberculin Skin Tests Positivity for US-Bound Refugee Children Tested Before, During, or After the Critical Interval

Variable	Overseas TST (N = 33 155)		Univariate Analysis: Crude OR (95% CI)	Multivariate Analysis^a^: Adjusted OR (95% CI)
	Positive TST Result, No. (%)	Negative TST Result, No. (%)		
Sex				
Male	1211 (7.2)	15 726 (92.9)	Reference	Reference
Female	1128 (7.0)	15 090 (93.0)	0.97 (0.89–1.06)	0.98 (.90–1.07)
Age at examination				
2–4 y	526 (4.9)	10 308 (95.1)	Reference	Reference
5–9 y	730 (5.8)	11 942 (94.2)	1.20 (1.07–1.34)	1.31 (1.17–1.48)
10–14 y	1083 (11.2)	8566 (88.8)	2.48 (2.22–2.76)	2.67 (2.39–2.99)
Tuberculosis contact				
Yes	83 (72.2)	32 (27.8)	35.36 (23.47–53.29)	25.41 (16.60–38.92)
No	2256 (6.8)	30 784 (93.2)	Reference	Reference
TST timing relative to vaccination with any live-virus vaccine^b^				
Before critical interval	838 (6.6)	11 943 (93.4)	0.91 (.83–.99)	0.63 (.55–.72)
During critical interval	65 (15.7)	349 (84.3)	2.40 (1.84–3.20)	2.40 (1.79–3.22)
After critical interval	1436 (7.2)	18 524 (92.8)	Reference	Reference

Abbreviations: CI, confidence interval; OR, odds ratio; TST, tuberculin skin test.

^a^The multivariate model includes all 4 variables shown in the table plus country of examination, not shown. Countries where <20 people had a positive TST were grouped together, collapsing the country value to “other” for 4% of individuals.

^b^Vaccination with ≥1 of the following: vaccine containing ≥1 of measles, mumps, or rubella vaccine; oral polio vaccine; or varicella vaccine. For a given vaccine, the critical interval is defined as 1–28 days following the last vaccination administered before the TST. If the TST occurred within this window, the child was tested during critical interval. If the TST occurred >28 days after the last vaccination, the child was tested after the critical interval. If the last vaccination occurred on the same day as the TST, the child was tested before the critical interval. Testing during the critical interval is not historically recommended. This TST model used the “after” group as the reference for the TST timing variable; when the “before” group is used as the reference, the adjusted OR for children vaccinated during the critical interval is 3.81 (95% CI, 2.80–5.18).

### Measles Vaccine

For the 25 430 children who received a measles vaccine rather than the MMR required for immigration, there was no significant difference in the adjusted odds of a positive IGRA between those tested during the critical interval and those tested after it (aOR, 0.62 [95% CI, .36–1.06]) or before it (1.30 [.83, 2.04]). There was also no significant difference in the adjusted odds of a positive TST between those tested during the critical interval and those tested after it (aOR, 0.25 [95% CI, .03–1.85]) or before it (<0.001 [<.001 to >999.999]) because 50% of the cells had expected counts <5.

## DISCUSSION

The recommendation to delay testing for tuberculosis infection until 4 weeks [10, 11] after receipt of live-virus vaccines is problematic for people immigrating to the United States, who are required to receive vaccinations and tuberculosis infection testing. The historical evidence supporting the delay was limited and therefore warranted additional investigation. Our analysis did not provide evidence that children who received a TST or IGRA within 1–28 days of receiving a live-virus vaccine were more likely to test negative than children tested after 28 days. In fact, we observed a small increase in positive results among children tested during the critical postvaccination interval, challenging the theory that vaccine-induced immune suppression causes false-negative results.

The historical studies reporting suppression of TST reaction following receipt of live-virus vaccines were limited in multiple ways. In the commonly quoted study by Starr and Berkovich from 1964 [4], the sample included only 6 children with tuberculosis disease who received the measles vaccine and were not also exposed to wild-type measles. That study did not involve the measles virus–containing vaccine or TST method currently used, and the results among those 6 children were variable. In the study by Berkovich and Starr from 1966 [5], involving OPV in children with tuberculosis disease, 7 of 20 children vaccinated for polio demonstrated suppressed TST response beginning at 4 weeks; however, in 4 of these 7 the suppression coincided with a significant viral illness suspected to be the cause. In addition, 3 of the 10 children who did not receive polio vaccine demonstrated similar suppression in this study. Also, of note, all children in both studies had tuberculosis disease, which has long been implicated in causing anergy [20]. There are no publications suggesting that IGRA is affected by live vaccines, yet the same recommendation is made to delay this test [10, 12].

Advocates of this delay have expressed concern of false-negative TST results caused by MMR vaccine leading to missed cases of tuberculosis disease, thus potentially resulting in death [21]. Numerous other factors cause immune suppression, including tuberculosis disease itself, so a negative TST or IGRA result should never be used to rule out tuberculosis disease; rather, signs and symptoms, chest radiography, cultures, and molecular tests should be used [12]. Even if MMR vaccine did increase the likelihood of false-negatives—a finding our analysis does not support—a negative TST/IGRA result should not result in a missed case of tuberculosis disease [15].

Even though TSTs and IGRAs have limited benefit in diagnosing tuberculosis disease, they are helpful in detecting latent tuberculosis infection (LTBI,) which can be treated on arrival to the United States. If result are negative during the overseas examination occurring >6 months before arrival in the United States, domestic guidance for refugee children recommends testing again shortly after arrival and perhaps testing later in life, depending on travel history and health factors [17, 22]. Not only can TST/IGRA results be falsely negative during the overseas examination, but exposures can occur in the final days before travel, in US communities, or during future travel. Thus, although a captured diagnosis of LTBI overseas is helpful, a missed diagnosis might be captured later during recommended follow up testing as part of routine primary care [22].

The surprising outcome of this analysis was the slightly higher, not lower, prevalence of positive results when the test was administered during the critical period. This effect was larger for TST than for IGRA and has not been previously documented in the literature. Although additional investigation is needed to confirm this finding, if discontinuing the recommended delay does lead to a small increase in the number of children with positive IGRA results, this might lead to more children being referred for further tuberculosis disease evaluation or receiving unnecessary LTBI treatment, resulting in adverse effects or other adverse events. However, given that this increase in positive results for IGRA is small, this might not represent a clinically significant finding.

There are multiple limitations to this analysis. Although country of examination, exposure to tuberculosis disease (ie, known contacts), and immigrant versus refugee status were controlled for, other unmeasured factors that caused the children to be tested during the recommended wait period might have contributed to an increased likelihood of having a positive test result. Some providers attempted to prevent testing during the critical period, and circumstances that required them to do so for some children are unclear and could represent an unknown confounder. A gold standard for LTBI testing that could be used for comparison does not exist. Inferences about a causal relationship between live-virus vaccines and increased likelihood of positivity for TST and IGRA are premature given the inherent limitations of observational data.

In summary, the original studies suggesting that administering TST during the 4 weeks after receipt of a live-virus vaccine could produce false-negative test results through vaccine-induced immune suppression are limited, as described above. This analysis does not support the supposition that a delay is needed before IGRA or TST testing to prevent false-negative results. The overseas medical examinations now require the use of IGRAs rather than TSTs, for which the positive effect was small. Removing this delay during the overseas medical examination could simplify operational processes, reduce waiting times, and provide more opportunities for vaccination. In conclusion, these findings do not support the need to delay testing with IGRA or TST after vaccination for US immigrants and refugees during their required medical examinations.

## Notes

***Author contributions.*** J. J. R. planned the project and wrote the initial draft and subsequent revisions of the manuscript. Z. W. conducted the analysis, wrote the initial draft of the methods and results sections, and reviewed and edited subsequent drafts of the manuscript. C. R. P. supervised and contributed to the project design, analysis, writing, and revisions of the manuscript.

***Financial support.*** The authors are federal employees. No funding was received to conduct this study.
